# Insights into the Draft Genome Sequence of a Haitian Variant *Vibrio cholerae* Strain Isolated from a Clinical Setting in Kerala, South India

**DOI:** 10.1128/genomeA.00843-17

**Published:** 2017-09-07

**Authors:** Lekshmi Narendrakumar, Karthika Suryaletha, Dinesh Reghunathan, Manoj Prasannakumar, Sabu Thomas

**Affiliations:** a Cholera and Biofilm Research Lab, Rajiv Gandhi Centre for Biotechnology, Trivandrum, Kerala, India; b University of Kerala, Kerala, India; c Genomics Core Facility, Rajiv Gandhi Centre for Biotechnology, Trivandrum, Kerala, India

## Abstract

We report here the draft genome sequence of a Haitian variant *Vibrio cholerae* strain, W4-13, isolated from Kerala, South India, possessing cholera toxin gene in chromosomes I and II. The sequence will be useful to achieve a profound understanding on its evolution, with emphasis on its pathogenesis and antibiotic resistance.

## GENOME ANNOUNCEMENT

India, being an area in which cholera is endemic, experiences periodic outbreaks of the disease even today. The appearance of evolved strains of *Vibrio cholerae*, the causative agent of cholera, and its dissemination are causes of great concern to all developing countries (1, 2). Major genetic traits of *V. cholerae* O1 Haitian isolates were previously reported (3).

The sequenced strain was isolated during a sporadic cholera outbreak in the Wayanad district of Kerala, South India, in 2013 from the stool sample of a diarrheal patient. The isolate was identified to be toxigenic, possessing all major virulence genes. The *ctxB* gene sequence was similar to that of the Haitian outbreak strains (*ctxB7*), and *rstB* had a genotype similar to those of the Kolkata variants. PCR analysis revealed that the strain possessed the cholera toxin gene in both chromosomes, unlike the Haitian variants reported so far. The isolate W4-13 was found to be resistant to trimethoprim, co-trimoxazole, streptomycin, and nalidixic acid by a disk diffusion assay. It also amplified the *strB* and *sul2* genes responsible for streptomycin and sulfamethoxazole, respectively. The above-mentioned features prompted us to sequence the strain to achieve a profound understanding on its evolution, with emphasis on its virulence and antibiotic resistance determinants.

Total genomic DNA was isolated using the Wizard genomic DNA purification kit (Promega), per the manufacturer’s protocol. The library was prepared using the Ion PGM library kit and loaded onto a 318 Chip version 2 provided by Ion Torrent (Life Technologies, Inc.). Sequencing by the Ion Torrent PGM platform yielded a total of 2,139,628 reads (40× genome coverage). The draft genome was assembled using SPAdes version 3.1 (4), which resulted in 123 contigs, with a total sequence length of 3,993,976 bp and an *N*_50_ of 56,569 bp. The longest contig was 159,256 bp in size. The strain showed a G+C content of 47.52%. The genome was annotated using NCBI Prokaryotic Genome Annotation Pipeline (PGAP) (5) and analyzed using the Rapid Annotations using Subsystems Technology (RAST) server (6). Resistance genes were predicted by ResFinder version 2.1 (7).

The annotation process identified 3,828 genes, 3,763 coding sequences, 791 pseudogenes, 553 subsystems, and 65 RNAs, including 9 rRNAs, 52 tRNAs, and 4 noncoding RNAs (ncRNAs). Genomic analysis revealed the presence of cholera toxin genes on both chromosomes. ResFinder detected an aminoglycoside gene, *strB*, and a sulfonamide resistance gene, *sul2*, with 100% identity, and an aminoglycoside gene (*strA*), phenicol resistance genes (*floR* and *catB9*), and a trimethoprim resistance gene (*dfrA1*), with 99% identity. Genes encoding multidrug efflux pumps and mobile element proteins were also identified. A detailed comparison of this strain to the prototype *V. cholerae* strains and Haitian outbreak strains will be presented in a future publication. Genomic information gathered from the comparative analysis will provide us a better understanding of the pathogenesis and resistance determinants of the evolved *V. cholerae* strains.

### Accession number(s).

The whole-genome shotgun project of the strain has been deposited at DDBJ/EMBL/GenBank under the accession number NIWX00000000. The version described in this paper is the first version, NIWX01000000.
